# The current status of lipoprotein (a) measurement in clinical biochemistry laboratories in the UK: Results of a 2021 national survey

**DOI:** 10.1177/00045632231210682

**Published:** 2023-11-08

**Authors:** Saleem Ansari, Robert D. Garmany Neely, Jules Payne, Jaimini Cegla

**Affiliations:** 1 Division of Diabetes, Endocrinology and Metabolism, 4615 Imperial College London, London, UK; 2 Lipids and Cardiovascular Risk Service, Department of Cardiology, Hammersmith Hospital, 8946 Imperial College Healthcare NHS Trust, London, UK; 3 Academic Health Science Network for the North East and North Cumbria, Newcastle Upon Tyne, UK; 4 14192 Heart UK, Maidenhead, UK

**Keywords:** Lp(a), lipoprotein(a), measurement of Lp(a), lipoprotein(a) measurement, standardisation, Lipids, Cardiovascular risk

## Abstract

**Background:**

Lipoprotein(a) (Lp(a)) is now established as a causal risk factor for cardiovascular disease (CVD) and accurate laboratory measurement is of pivotal importance in reducing Lp(a) associated risk. The consensus statement by HEART UK in 2019 included recommendations to improve standardisation of clinical laboratory measurement and reporting of Lp(a).

**Methods:**

A 16 question, electronic audit survey was circulated to 190 accredited clinical biochemistry laboratories to assess the adoption of these recommendations in the UK.

**Results:**

Responses were received from 65 of 190 laboratories (34%). Only 5 (8%) did not offer Lp(a) measurement. Of those providing the test, 23% (n = 14) offered an in-house service (IHS), the remaining laboratories (77%; n = 46) used an external referral service (ERS). The majority (10 of 14 or 71%) of IHS laboratories responded with details of their method, stating whether it minimised sensitivity to the effect of Lp(a) isoform size and used calibrators certified for traceability to the WHO/IFCC reference material, however, only a minority ERS laboratories (13 of the 46 or 28%) were able to specify the method used by their referral laboratory. Of the laboratories who specified their reporting units, 6 of 10 IHS and 7 of 23 ERS laboratories reported in nmol/L. Among the 60 laboratories who responded, the HEART UK recommendations appear to have been adopted in full by only 3 IHS laboratories.

**Conclusions:**

Further efforts are needed to standardise the measurement and reporting of Lp(a) so that results and interpretation are comparable across clinical biochemistry laboratories in the UK.

## Introduction

Lipoprotein(a) (Lp(a)) is an independent risk factor for atherosclerotic cardiovascular disease (ASCVD) and calcific aortic valve stenosis.^1^ The cardiovascular risk conferred by Lp(a) in large epidemiological studies^2,3^ has, however, been inconsistent and often underestimated owing to shortcomings of the commercially available immunoassays used for Lp(a) measurement. In contrast, genome-wide association^4^ and Mendelian randomisation studies^5^ consistently provide evidence for a causal role of Lp(a) in ASCVD, confirmed by the more robust immunoassay methods used in more recent studies.^6^

**Figure 1.**
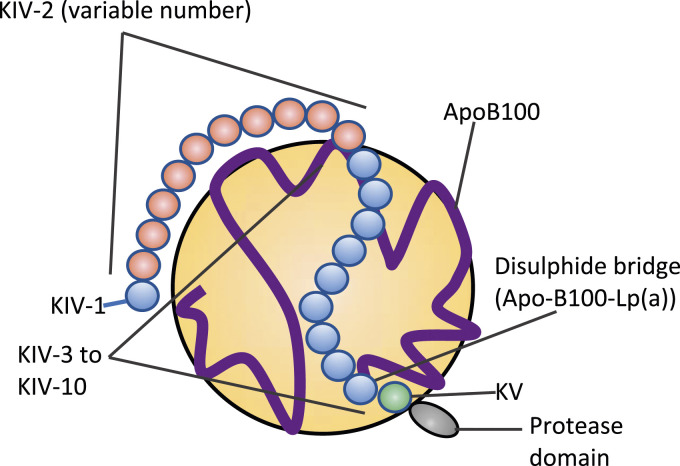
Lipoprotein (a) structure. Lipoprotein(a) contains a single molecule of Apolipoprotein(a) and ApoB100 (blue ribbon) around the low-density lipoprotein-like portion (large yellow circle) of this particle. Apolipoprotein(a) contains 10 classes (coloured balls) of kringle modules, designated KIV-1-10, KV and a protease domain. The variable number of KIV-2 modules forms the basis for the observed isoform size heterogeneity of apolipoprotein(a).

Lp(a) is an LDL-like particle containing a single apolipoprotein B100 covalently bound to a single apolipoprotein(a) (apo(a)) by a disulphide bond (Figure 1). The variable molecular mass of apo(a) is genetically determined by the inheritance of >40 allelic variants of *LPA* which define the polypeptide sequences of isoforms of different molecular mass, predominantly due to the number of Kringle IV type 2 repeats, which ranges from <3 to >40 in number.^7^ There is a well-established inverse relationship between the isoform size of Lp(a) and concentration in plasma. Smaller isoforms are associated with higher concentrations which confer increased CVD risk. Indeed within populations, Lp(a) concentrations can vary by 1000-fold.^8^ For many years, plasma concentrations of Lp(a) have been reported in mass units (mg/dL) but this is fundamentally unsound because the variable lipid content of Lp(a) (cholesterol, phospholipids, triglycerides) also influences the mass of this LDL-like particle and well-designed immunoassays measure only apo(a), thereby reflecting particle number, not particle mass. The most appropriate units of measurement for Lp(a) are therefore nmol/L and conversion of results between mass and molar units is not recommended.^9^

The presence of Kringle IV type 2 repeats in apo(a) presents one of several analytical challenges for Lp(a) measurement. The antibodies used against apo(a) are usually polyclonal and can bind multiple KIV-2 repeats. This overestimates Lp(a) plasma concentrations in individuals with large isoforms and underestimates the concentrations in those with small isoforms.^10^ Because of the heterogeneity in apo(a) size and the presence in most individuals of two different, genetically determined apo(a) isoform sizes, standardisation using a single calibrant material is impossible. In recent years, international organisations (IFCC and WHO) and commercial assay manufacturers have collaborated to improve the standardisation and accuracy of Lp(a) measurements. A well-characterised reference material (WHO/IFCC SRM-2B) is now available to assign molar values to calibrators.^11^ In addition, some commercially available assays use Denka-based reagents (Denka Seiken Co. Ltd, Japan) which minimise isoform-dependent bias by using a 5- point calibrator, consisting of a range of Lp(a) isoforms. The development of an appropriate reference material alongside commercially available immunoassays that minimise sensitivity to the effect of isoform size now offers an accurate and reliable approach to the graded assessment of Lp(a) associated ASCVD risk guided by measurements of Lp(a) particle number.^9^

In their consensus statement on Lipoprotein(a), HEART UK identified those patients at high risk of ASCVD in whom Lp(a) should be measured and made recommendations regarding laboratory measurement and reporting of Lp(a) results, in keeping with the above principles, as developed by international organisations (Table 1). However, to date, the adoption of these improved procedures in the UK has not been reported. We therefore undertook a national, questionnaire-based survey to better understand the status of Lp(a) analysis and reporting by clinical biochemistry laboratories in the UK.

**Table 1.** Recommendations regarding laboratory measurement of lipoprotein (a) according to the HEART UK consensus statement (2019).

Clinical indications
a) Serum Lipoprotein(a) concentrations should be measured using a method where the effect of isoform size has been minimised using appropriate antibodies with calibrators certified for traceability of Lp(a) values to the WHO/IFCC reference material
b) Results should be expressed in nmol/L of Lp(a) particles
c) Conversion of mass units to molar units or vice versa introduces inaccuracy and should be discouraged
d) Currently only assays based on Denka reagents with calibrators traceable in nmol/L to WHO/IFCC reference material can be recommended

## Methods

A short, electronic survey comprising 16-questions (Supplemental Table 1) was designed on a standard commercial platform (Survey Monkey) and circulated to Association of Clinical Biochemistry mailbase subscribers working in clinical biochemistry laboratories in the UK. Responses were collected between July and October 2021. The survey consisted of four parts. The first part asked questions to identify their laboratory and the type of Lp(a) service they provided (5 questions). The second part addressed methodological details such as the instruments used, whether the assays minimised sensitivity to the effect of isoform size and traceability to WHO/IFCC reference material (4 questions). The third part addressed post-analytical considerations including units reported and any conversions applied, reference ranges and/or clinical action limits added to reports (3 questions). The fourth and final part sought to determine what, if any additional, information or comments are included on reports to assist interpretation and to identify the specialities from which requests for serum Lp(a) measurements are accepted. Response data were exported into a spreadsheet for manual data sorting and cleaning including identification and elimination of duplicates entries. Responses from laboratories providing an in-house service (IHS) and from ‘send-away’ labs, that is, those using an external referral service (ERS) provider, were analysed separately and responses received from IHS and ERS laboratories were cross-referenced. Results have been reported as n or % unless otherwise stated. All graphs have been produced using Prism Graphpad Version 9.

## Results

### Part 1 Lipoprotein(a) service provision

**Figure 2.**
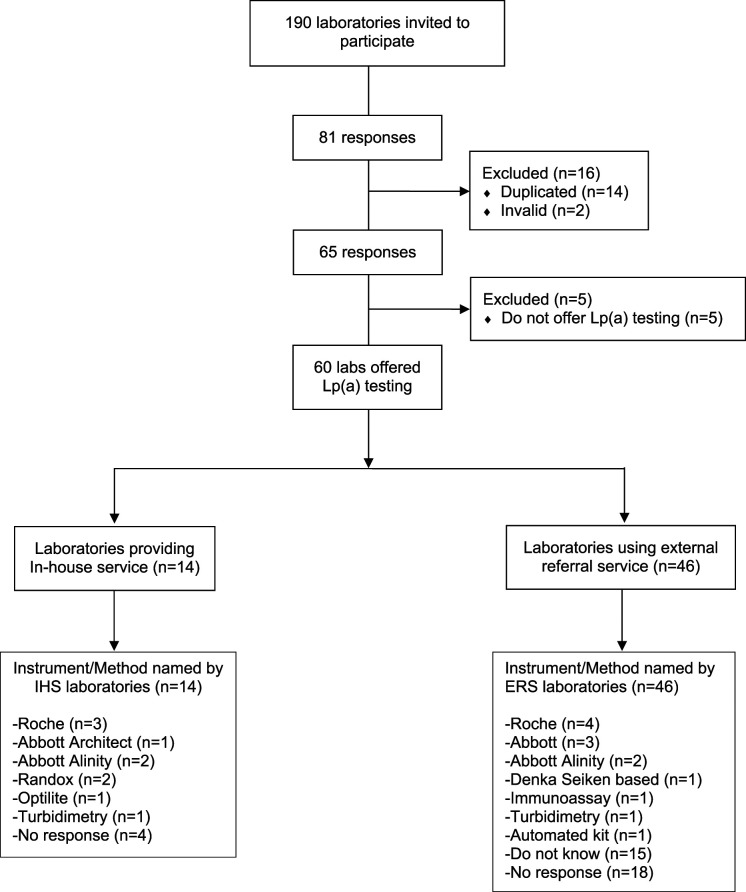
Laboratories that responded to our survey categorised as in-house service (IHS) versus external referral service (ERS) providers and the instruments/methods used to measure lipoprotein (a).

After exclusion of duplicates and invalid responses, responses were obtained from 65 of 190 accredited clinical biochemistry laboratories in the UK (34% response rate) (Figure 2). Almost all respondents were from NHS laboratories (92%) with a small number coming from joint private-NHS partnership (3 sites) or private laboratories (2 sites). Lp(a) measurement was offered by 92% of responding laboratories (n = 60), only 8% did not (n = 5). Fourteen laboratories performed in-house Lp(a) testing (23%) and the remaining 46 laboratories (77%) sent their samples to named external referral laboratories (n = 12), only two of which did not submit a response to the survey. A regional breakdown of these IHS and ERS laboratories is presented in Supplemental Table 2.

### Part 2 Lipoprotein(a) method details

**Figure 3.**
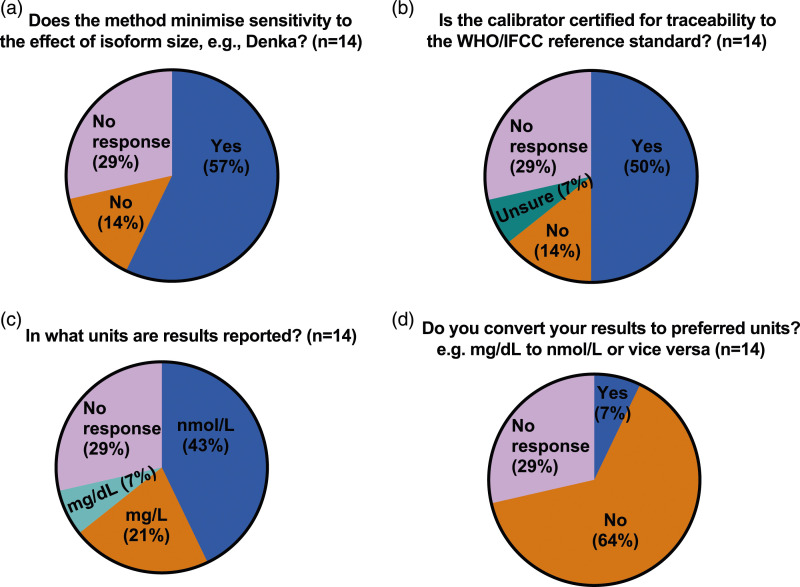
Pie chart (a–d) showing responses to questions relating to the measurement of lipoprotein(a) from in-house service (IHS) provider laboratories in the UK.

**Figure 4.**
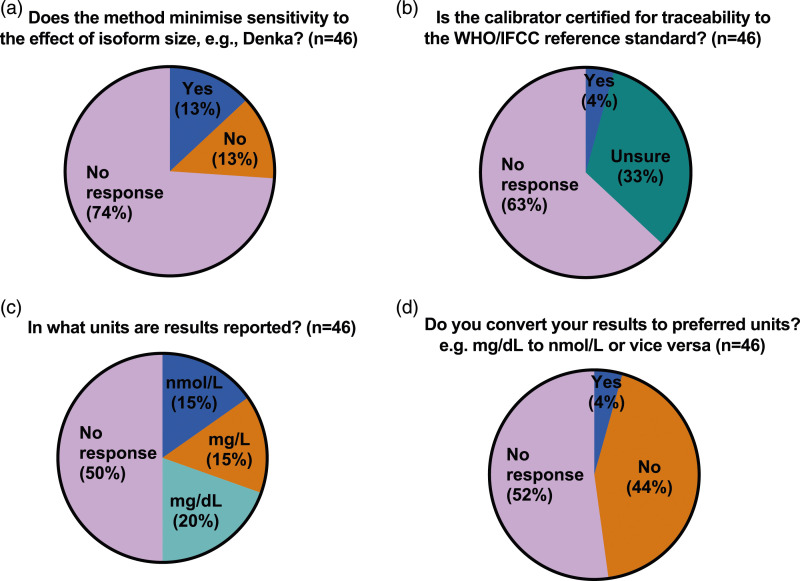
Pie chart (a–d) showing responses to questions relating to the measurement of lipoprotein(a) from external referral service (ERS) provider laboratories in the UK.

While the majority (10 of 14 or 71%) of IHS laboratories responded with details of their method, only 3 specified both platform and reagents, 4 specified the instrument/platform only, 2 specified only the reagents used and one gave only generic information regarding the type of method (turbidimetry). The 4 IHS laboratories which did not provide any method details also skipped all the remaining questions and sections of the questionnaire. In contrast, while most ERS laboratories (38 of 46) were able to provide the name of their referral laboratory, only 13 (28%) were able to specify the method used (9 specified the instrument/platform only, 1 specified only the reagents used and 3 gave only generic information regarding the type of method), 15 were not sure or the method was not known, including one giving incorrect information (specified an instrument/platform not used by the referral laboratory they named earlier in the questionnaire) and 18 gave no response. (Figure 2). When asked if their method minimised sensitivity to the effect of effect of Lp(a) isoform size, 2 IHS laboratories responded ‘No’ and 8 responded ‘Yes’ but 2 of the latter (both Roche method) gave duplicate responses with conflicting answers. When asked if their calibrator was certified for traceability to the WHO/IFCC reference standard the same 10 IHS laboratories responded, of whom 7 responded ‘Yes’, 2 responded ‘No’ and one respondent, who gave only generic information regarding the type of method answered ‘Unsure’ (Figures 3(a) and (b)). When ERS laboratories were asked if their method minimised sensitivity to the effect of effect of Lp(a) isoform size, only 12 of the 46 responded, 6 with ‘No’ and 6 with ‘Yes’. In 2 cases (one answering ‘Yes’ and one ‘No’), the ERS laboratory response did not correspond to the answer given by their referral laboratory. When ERS laboratories were asked if their calibrator was certified for traceability to the WHO/IFCC reference standard, only 17 of 46 laboratories responded, 15 of which were ‘unsure’ and only 2 responded ‘yes’, again in one case the ERS laboratory response did not correspond to the answer given by their HIS referral laboratory. (Figures 4(a) and (b)).

### Part 3 Lipoprotein(a) units and reporting

Only 10 of the 14 IHS laboratories specified their units for reporting lipoprotein(a) measurements. Of these, 6 reported in molar units (nmol/L), 4 in mass units (3 laboratories mg/L; one laboratory mg/dL) and one reported using a conversion factor to convert to their preferred units (mg/dL). In contrast, of the 23 ERS laboratories who responded, the majority specified mass units (7 laboratories mg/L; 9 laboratories mg/dL) the remaining 7 laboratories reported in molar units (nmol/L). Of these, only two (one private and one NHS) reported using a conversion factor to convert to their preferred units (nmol/L and mg/L respectively).

Only 10 of the 14 IHS laboratories specified what reference range values or clinical action limits they added to their reports. Of these, 3 laboratories who reported results in nmol/L also used the HEART UK consensus recommendations to grade cardiovascular risk based on Lp(a) concentration (32–90 nmol/l minor; 90–200 nmol/l moderate; 200–400 nmol/l high; >400 nmol/l very high).^12^ A single threshold value for elevated risk was used by 2 laboratories (>125 nmol/L and >300 mg/L), while another laboratory reporting in nmol/L specified two threshold values (>75 nmol/L increased risk; >120 nmol/L high risk). Low risk/normal threshold values were specified by 3 laboratories (<75 nmol/L; <300 mg/L; <30 mg/dL), with only one laboratory specifying a reference range (0–300 mg/L). Of the 46 ERS laboratories, the 22 who responded to this question gave a wide variety of answers, including 3 who quoted the HEART UK consensus recommendations, 3 who used the values quoted by their reference laboratory, 6 adding reference ranges in mg/dL (normal less than 30 mg/dL), 3 adding reference ranges in mg/L (normal less than 300 mg/L), 2 who specified none and one respondent who quoted ApoA1 reference intervals in error. The responses given by IHS and ERS laboratories are summarised in Table 2. Additional comments or interpretative information (unspecified) were added by only 3 laboratories (1 IHS provider and 2 ERS laboratories). Only one laboratory (a private ERS laboratory) reported that they apply a correction for Lp(a)-C in their LDL-C calculation, despite reporting Lp(a) in molar units.

**Table 2.** Lipoprotein (a) reference ranges reported by in-house service (IHS) provider and external referral service (ERS) provider laboratories in the UK.

	Laboratories	
Reference range/Action limits	IHS (n = 10)	ERS (n = 22)
HEART UK recommendations (%)	30	14
<75 nmol/L (%)	20	14
>90 nmol/L (%)	0	5
>120 nmol/L (%)	10	0
>125 nmol/L (%)	10	0
>/<30 mg/dL (%)	10	28
<300 mg/L (%)	30	14
As quoted by referral laboratory (%)	N/A	14
None (%)	0	10
*Total (%)*	100	100

### Part 4 Requesting specialities

From the total of 60 laboratories who responded to the questionnaire, 32 laboratories (10 IHS and 22 ERS) reported the clinical specialities from whom they accept Lp(a) requests, as summarised in Table 3. Of the 10 IHS laboratories who responded, 8 accepted requests from the widest range of specialities, specifically from lipid specialists, cardiologists, stroke physicians, diabetes/endocrinology, or any hospital consultant as well as GPs. The other 2 sites restricted requests to lipid specialists and diabetes/endocrinology and lipid specialists and cardiologists, respectively. The range of specialities from which requests were accepted were more restricted in ERS laboratories, with 12 restricting requests to lipid specialists only, 4 to lipid specialists and cardiologists. Only 7 of 22 sites accepted requests from the widest range of specialities (lipid specialists, cardiologists, stroke physicians, diabetes/endocrinology) or any hospital consultant and only 6 accepted requests from GPs.

**Table 3.** Lipoprotein (a) requests according to speciality from in-house service (IHS) provider and external referral service (ERS) provider laboratories in the UK.

Specialities	IHS (n = 10)	ERS (n = 22)	Overall (n(%))
Lipid	10	22	32 [100]
Cardiology	10	12	22 [69]
Any hospital consultant	9	8	17 [53]
Vascular	8	7	15 [47]
Stroke	8	6	14 [44]
Diabetes/endocrine	9	5	14 [44]
Primary care	7	5	12 [38]

## Discussion

The HEART UK consensus statement on lipoprotein(a), published by in 2019, was based on the evidence that Lp(a) is an independent, causal, but largely unmeasured risk factor for ASCVD, and included recommendations for its measurement reporting in clinical laboratories.^12^ In addition to specifying requirements for accurate Lp(a) measurements, expressed in molar units, the statement recommended the categories of individuals in whom Lp(a) should be measured and proposed a graded approach to interpretation of Lp(a)-associated risk, as determined by the serum concentration of lipoprotein(a) particles (32–90 nmol/l minor; 90–200 nmol/l moderate; 200–400 nmol/l high; >400 nmol/l very high risk). To assess the adoption of these recommendations in the UK, a 16-question survey was sent to clinical biochemistry laboratories via the ACB Mailbase. Based on responses from 65 (38%) of 190 accredited laboratories, only 5 of which did not offer Lp(a) measurement, it is clear that considerable variation is to be found in the analysis and reporting of Lp(a) among both laboratories providing an in-house service (IHS) and the larger number (46 of 60 or 76%) of laboratories using external referral services (ERS). Although over 50% of IHS laboratories use Denka-based reagents for their assays, most ERS laboratories were unaware if they were using assays with calibration traceable to the WHO/IFCC reference material and most reported Lp(a) in mass units (mg/dL or mg/L). Overall, only 3 of 60 laboratories, all IHS providers, appeared to have implemented the HEART UK recommendations in full.

A compelling body evidence supports a causal role for lipoprotein(a) in atherosclerotic cardiovascular disease (ASCVD) and calcific aortic valve stenosis (CAVS) as demonstrated by large epidemiological,^13^ genome-wide association^4^ and Mendelian randomisation studies.^5^ As a result, therapeutic strategies to lower Lp(a) (using injectable therapies based on antisense oligonucleotides^14^ and small interfering RNA molecules,^15^ followed recently by an orally active therapy^16^) have progressed rapidly with publication of clinical outcomes studies anticipated in the next 3–5 years. If favourable clinical outcomes are observed with these Lp(a) lowering therapies, then approvals from the European Medicines Agency (EMA), Medicines and Healthcare products Regulatory Agency (MHRA) and the National Institute of Health and Care Excellence (NICE) will be sought, and to support their implementation clinical laboratories in the UK will need to be ready to provide accurate, standardised, Lp(a) measurements and interpretive information.

Although these specific therapies to lower Lp(a) serum concentrations are not yet available, the measurement of Lp(a) is required for personalised risk assessment and to achieve meaningful reduction of Lp(a)-associated ASCVD risks by other means^12^; at present this means laboratory-based testing. Measurement of Lp(a) in an accredited laboratory is, however, no guarantee of reliable results as widely used assays on platforms from major manufacturers do not meet the HEART UK recommendations. Concerns regarding Lp(a) assays led many UK laboratories to discontinue their provision of an Lp(a) service following the publication in 2009 of a HEART UK sponsored comparative study of Lp(a) measurements in 36 UK laboratories using 12 different assay methods.^17^ This study showed extreme between-laboratory and between-assay variation, enough to render Lp(a) results meaningless, and called for a moratorium on the use of Lp(a) concentration as a clinical tool until the problems inherent in the assays could be resolved. Accurate measurement of Lp(a) can only be achieved if the assay is isoform insensitive so that one particle of high molecular weight Lp(a) produces the same signal as one particle of low molecular weight Lp(a).^10^ Although none of the current commercially available Lp(a) assays are truly isoform insensitive, assays using Denka-based reagents (Denka Seiken Co. Ltd, Japan) and calibration protocols are currently the least isoform sensitive because they employ five independent calibrators across the reporting range and each calibrator contains a suitable distribution of apo(a) isoforms traceable in nmol/L to the WHO/IFCC reference material.^9^ This approach better matches calibrants with patient samples and has acceptable bias compared with the gold standard ELISA method.^10^ Laboratory providers need to be confident that their assays employ suitable reagents (e.g. Denka-based) and calibrators traceable to the WHO/IFCC reference material which is reported in molar units (nmol/L).^11^ The Northwest Lipid Research Laboratory (University of Washington) provides a certification process for laboratories that evaluates Lp(a) assay performance and traceability to WHO/IFCC SRM-2B by comparing the Lp(a) values to those obtained by the reference monoclonal antibody-based ELISA method.^9^ In the present survey, over 50% of IHS laboratories reported using Denka-based reagents.

Until recent years measurement of lipoprotein(a) had been largely the preserve of lipid specialists, many of whom as Chemical Pathologists have had a decision-making role in their clinical biochemistry laboratory service provision. However, even those laboratories serving lipid clinics may not generate sufficient numbers of requests to make in-house provision of Lp(a) measurement a viable proposition. It comes as little surprise to find that the majority of laboratories responding to our survey referred their samples to an external laboratory. The choice of method, units of measurement, interpretative information including reference intervals and comments on reports will then, largely, be determined by the referral laboratories, each of which may thereby influence the practice of numerous ‘send-away’ laboratories. In our survey, the IHS laboratories were more likely to respond to questions regarding method details such as isoform sensitivity and traceability of calibrators to international reference preparation, and although a higher proportion reported Lp(a) in molar units, only a minority followed the HEART UK recommendations to report graded risk.^12^ Those laboratories using an external referral service (ERS) were less often able to answer questions about method details and were more likely to report Lp(a) in mass units, quoting reference ranges or dichotomous cut-off values (30 mg/dL or 300 mg/L) derived from older literature^18,19^ and manufacturer’s product information. Until the larger IHS laboratories, accepting referrals from smaller ERS laboratories, ensure their Lp(a) analysis and reporting practices follow the HEART UK recommendations, this situation is unlikely to improve. Of course, the choice of method is, for many laboratories, influenced by their choice of a single manufacturer reagent and platform deal under a laboratory managed service contract, which may restrict their ability to choose third party reagents which would meet the recommendations of HEART UK and other expert bodies.

The principle that Lp(a) measurement should be considered at least once in each adult’s lifetime has been promoted by European and North American clinical practice guidelines including those from European Atherosclerosis Society.^20–24^ The European Cardiology Society guideline aims to identify those with very high Lp(a) values >430 nmol/L (99th percentile) who have a lifetime cardiovascular risk equivalent to patients with heterozygous familial hypercholesteraemia.^21^ HEART UK has advocated a more selective approach, targeting those high risk individuals most likely to benefit from measures to reduce Lp(a)-associated CVD risk.^12^ In keeping with this approach, recently published National Clinical Guideline for Stroke, developed in association with NICE, recommends Lp(a) measurement in people with ischaemic stroke or TIA of presumed atherosclerotic cause below 60 years of age.^25^ Even implementation of these more conservative guidelines will require making Lp(a) measurements available to a wider range of health professionals than is currently the case. In the present survey, the range of specialities from which Lp(a) requests were accepted was more restricted by ERS laboratories, less than a third accepting requests from the widest range of specialities including stroke physicians and GPs, compared to the 80% of the responding IHS laboratories who offered this wider access. The reasons for this apparent difference were not captured by our survey and but possible explanations, which may include local commissioning, cost constraints and clinician awareness of guidelines, deserve further exploration.

When clinical decisions are to be made based on Lp(a) results, both accurate measurement and correct interpretation are required. The present survey highlighted extreme heterogeneity of Lp(a) reference ranges and interpretive information quoted by biochemistry laboratories, and this most likely reflects a range of factors including historical reporting practices, underlying differences in platforms, assays, reagents and calibrators and recapitulates the urgent need for standardisation and harmonisation to assist laboratorians, clinicians and patients, in a similar fashion to the standardisation and harmonisation of glycated haemoglobin.^26^ To support the development of their consensus statement, the HEART UK Medical and Scientific Committee were granted access to data from 14,000 participants in the ongoing Copenhagen General Population Study who had Lp(a) measured using the Roche Cobas Gen II assay, based on the Denka Seiken reagents. With this dataset, they were able to grade Lp(a) associated CVD risk on the basis of population percentile distributions, as follows: 32–90 nmol/l minor; 90–200 nmol/l moderate; 200–400 nmol/l high; >400 nmol/l very high.^12^ Although reporting in mass units predominated overall among the respondents to the present survey, encouragingly over 50% of IHS laboratories reported in molar units and quoted the HEART UK clinical action limits. A cut-off >90 nmol/L represents the 80th percentile for Lp(a) levels based on the Copenhagen Study, which is often used as a basis for the recommendation of a single cut-off (>50 mg/dL),^27^ but it should be noted that this dataset derives predominantly from a Danish Caucasian population and establishing clinically relevant Lp(a) cut-off values in a range of ethnicities is an area that requires further work. Other molar cut off values of >120 nmol/L and 125 nmol/L were reported separately by two laboratories, and the latter of these two thresholds is in keeping with US guidelines and approximately corresponds with the 90th percentile of the general population.^20^ Clearly, there are differences in the Lp(a) thresholds used to grade cardiovascular risk, not only in the present survey but also in national/international guidelines, but the reporting of graded risk, determined by Lp(a) concentration in nmol/L, as advocated by HEART UK appears to be the most accurate reflection of the continuous association of increasing Lp(a) concentrations with the risk of ASCVD and CAV. In parallel with efforts to harmonise Lp(a), analysis and reporting of routine lipid profiles would also benefit from better standardisation, specifically consensus on the measured parameters included (total cholesterol, HDL-cholesterol and triglycerides), calculated variables including non-HDL-cholesterol, LDL-cholesterol and Total:HDL-C ratio, and the requirements for fasting, as unacceptable interlaboratory differences have previously been reported.^28,29^

There are limitations of the present survey. Firstly, with an overall response rate of only 38%, the survey may not be truly representative of accredited UK laboratories and, in many cases, the responses did not include all the survey questions. Disappointingly, several of the larger IHS laboratories, to whom many laboratories send samples as an ERS provider, failed to a respond fully or did not respond at all. Laboratories not offering Lp(a) measurement may have ignored the invitation and thereby have been under-represented, however, they would also have been able to complete their response more quickly than others. Secondly, in order to minimise the complexity of the questionnaire, some aspects were not addressed such as organisational questions relating to workload, laboratory policy, costs and commissioning as well as practical barriers to the implementation of traceable molar assays. In order to address the current heterogeneity in Lp(a) measurement and reporting identified by this survey, better understanding of these important aspects will be required to develop an effective, collaborative approach to harmonisation of laboratory practice. One approach may be to invite IHS and ERS providers to a joint clinical and laboratory task force on the measurement of Lp(a), to increase stakeholder engagement and promote implementation of Lp(a) measurements in clinical practice as recommended in the National Clinical Guideline for Stroke^25^ and as may be recommended in future by NICE technology appraisals.

In conclusion, despite the urgent need to standardise and harmonise Lp(a) assays in the UK due to its clinical application in personalised cardiovascular risk assessment^12^ and rapid progress in the development of novel, specific therapies that can potently lower serum Lp(a) concentrations,^14–16^ clinical biochemistry laboratories in the UK still have a have much do to achieve these goals. The recommendations regarding Lp(a) measurement and reporting made in the 2019 HEART UK consensus statement have now been substantially adopted by the majority of in-house service providers who accept external referrals, but the majority of laboratories overall still report Lp(a) in mass units without evidence of traceability to the WHO/IFCC reference assay. Collaborative effort is needed to standardise the analysis and reporting of Lp(a) so that results are harmonised and interpretation comparable across all clinical biochemistry laboratories in the UK.

## Supplemental Material

Supplemental Material - The current status of lipoprotein (a) measurement in clinical biochemistry laboratories in the UK: Results of a national survey Supplemental Material for The current status of lipoprotein (a) measurement in clinical biochemistry laboratories in the UK: Results of a national survey by Saleem Ansari, R Dermot G Neely, Jules Payne and Jaimini Cegla in Annals of Clinical Biochemistry
