# Supplementary material for: The current status of lipoprotein (a) measurement in clinical biochemistry laboratories in the UK: Results of a 2021 national survey
Source: Ann Clin Biochem. 2023 Nov 8;61(3):195–203. doi: 10.1177/00045632231210682 (PMC11080397; doi:10.1177/00045632231210682)
Supplement: Supplemental Material - The current status of lipoprotein (a) measurement in clinical biochemistry laboratories in the UK: Results of a national survey [file sj-pdf-1-acb-10.1177_00045632231210682.pdf]

**Response rates per question in lipoprotein(a) survey**	**In-house laboratories that measure Lp(a)** (n=14)	**Sendaway laboratories that measure Lp(a)** (n=46)	**All laboratories that measure Lp(a)** (n=60)
1. Name of Lab (%)	100	96	97
2. Location (%)	100	100	100
3. Type of provider: NHS/Private/Joint partnership (%)	100	100	100
4. Does your laboratory offer Lp(a) a service for measurement? Y/N (%)	100	100	100
5. If Y to 4, is this assay performed in house? Y/N (%)	100	100	100
6. If Y to 5, what method does your laboratory use? Free text (%)	71	61	63
7. If N to 5, who is your referral lab and what method does your referral laboratory use? Free text (%)	0	83	N/A
8. Does the method minimise insensitivity to the effect of isoform size e.g. Denka? Y/N/ Not sure (%)	71	26	37
9. Is the calibrator certified for traceability to the WHO/ IFCC reference standard? Y/N/ Not sure (%)	71	37	45
10. In what units are the results	71	50	55

expressed? nmol/L or mg/dL or mg/L or g/L? (%)			
11. Do you convert results to your preferred units (e.g. mg/dL to nmol/L or vice versa) before reporting? Y/N (%)	71	48	53
12. What, if any reference range values or clinical action limits do you add to your reports? Free text (%)	71	48	53
13. Do you add any other information to your reports to assist interpretation/action on results (e.g. assay manufacturer, isoform dependency, re-test interval) (%)	71	48	53
14. Does your laboratory apply a correction for Lp(a)-C in LDL-C calculation? Y/N (%)	71	50	55
15. If Y to correction, what correction factor/formula do you use? (%)	0	0	0
16. What specialities do you accept Lp(a) requests from? (%)	71	48	53

Supplementary Table 1. Response rate for survey questions from in-house service (IHS) provider and external referral service (ERS) provider laboratories in the UK.

Region	IHS laboratories(n)	ERS laboratories (n)	No Lp(a) service (n)	All (n)
Scotland	1	5	0	6
Northern Ireland	1	5	0	6
Wales	1	2	0	3
North West	1	7	1	9
North East	2	1	0	3
Yorkshire and the Humber	0	5	1	6
West Midlands	1	6	1	8
East Midlands	0	3	0	3
East of England	0	2	1	3
South West	2	4	0	6
Greater London	3	0	0	3
South East England	2	6	1	9
Total	14	46	5	65

Supplementary Table 2. Clinical biochemistry laboratories that responded to our survey according to UK region. In-house laboratories providing in-house service (IHS) for lipoprotein(a) testing and laboratories using an external referral service (ERS) or no service.
